# Dependence on Cardiac Contractility Modulation Therapy: Loss of Continuous and Effective Modulation as a Cause of Heart Failure Exacerbation

**DOI:** 10.7759/cureus.111070

**Published:** 2026-06-18

**Authors:** Krzysztof Kaczmarek, Adam Wojtaszczyk, Pawel Ptaszynski, Jerzy K Wranicz, Iwona Cygankiewicz

**Affiliations:** 1 Department of Electrocardiology, Medical University of Lodz, Lodz, POL

**Keywords:** cardiac contractility modulation, cardiac rhythm devices, heart failure, implantable cardiac defibrillator (icd), transvenous lead extraction (tle)

## Abstract

Cardiac contractility modulation (CCM) is a device-based therapy for patients with heart failure, reduced left ventricular ejection fraction (LVEF), and narrow QRS who are not candidates for cardiac resynchronization therapy. CCM delivers non-excitatory impulses during the absolute refractory period, improving myocardial contractility without increasing oxygen consumption. We report a 58-year-old man with dilated cardiomyopathy and persistent New York Heart Association (NYHA) class III symptoms despite optimal therapy who underwent CCM implantation. The patient initially showed significant clinical improvement, with increased LVEF and functional status. However, several years later, he developed clinical deterioration associated with intermittent loss of effective CCM delivery. Surgical revision revealed insulation defects in all three leads, likely due to mechanical interactions. Lead replacement restored CCM function, resulting in marked and sustained improvement in exercise capacity, LVEF, and N-terminal pro B-type natriuretic peptide over long-term follow-up. This case highlights the importance of continuous and effective CCM delivery, as well as the need for careful long-term monitoring of device integrity, particularly in patients with multiple intracardiac leads.

## Introduction

Heart failure (HF) remains one of the most significant clinical challenges of the 21st century. Similar to other cardiovascular diseases, the incidence and prevalence of HF are increasing worldwide. Variations in the distribution of risk factors contribute to a disproportionately higher disease burden in specific populations [1]. Guideline-directed medical therapy (GDMT) constitutes the cornerstone of HF management [2]. In symptomatic patients with prolonged QRS duration and bundle branch block, cardiac resynchronization therapy (CRT) may provide significant symptomatic relief. However, many patients do not meet CRT eligibility criteria or fail to respond. For this population, cardiac contractility modulation (CCM) represents a potential device-based therapeutic option [3].

CCM delivers high voltage (~5 V), biphasic, non-excitatory impulses to the right ventricular septum during the absolute refractory period, enhancing myocardial contractility without inducing depolarization or increasing oxygen consumption [4]. Its mechanism involves improvement in intracellular calcium handling, including modulation of ryanodine receptor (RyR2), SERCA2a activity, and phospholamban phosphorylation [5,6]. CCM also enhances phosphorylation of contractile proteins such as troponin I and myosin-binding protein C, contributing to improved systolic function [6].

Experimental studies suggest that CCM may reverse maladaptive myocardial remodeling by restoring titin expression and reducing fibrosis via modulation of matrix metalloproteinases and TGF-β1/Smad3 signaling pathways [7]. These effects translate into improved ventricular function, reduced HF symptoms, and increased exercise capacity.

Currently, CCM is approved for patients with LVEF 25-45%, QRS duration <130 ms, and New York Heart Association (NYHA) class III symptoms [8-10].

## Case presentation

**Figure 1:**
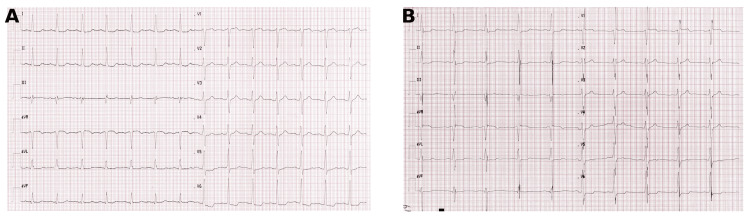
A. ECG before CCM implantation. B. ECG after CCM implantation. CCM stimulation spikes are visible CCM: Cardiac contractility modulation

We report a case of a 58-year-old male with hypertension and dilated cardiomyopathy who underwent CCM implantation in 2015. At baseline, the patient had LVEF 20%, sinus rhythm, narrow QRS (115 ms), and NYHA class III symptoms despite GDMT (Figure 1). He declined implantable cardioverter-defibrillator (ICD) therapy but accepted CCM as a symptomatic treatment option.

**Figure 2:**
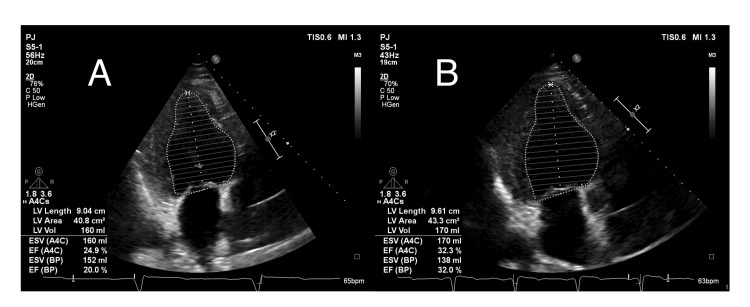
A. Left ventricle ejection fraction before CCM implantation. B. Left ventricle ejection fraction after CCM implantation CCM: Cardiac contractility modulation

Following implantation of an Impulse Dynamics Optimizer IVs system with three leads, the patient experienced marked clinical improvement. At three months, NYHA class improved to II, LVEF increased to 32%, and left ventricular end-diastolic volume decreased significantly (Figure 2). Device interrogation confirmed appropriate CCM delivery.

**Figure 3:**
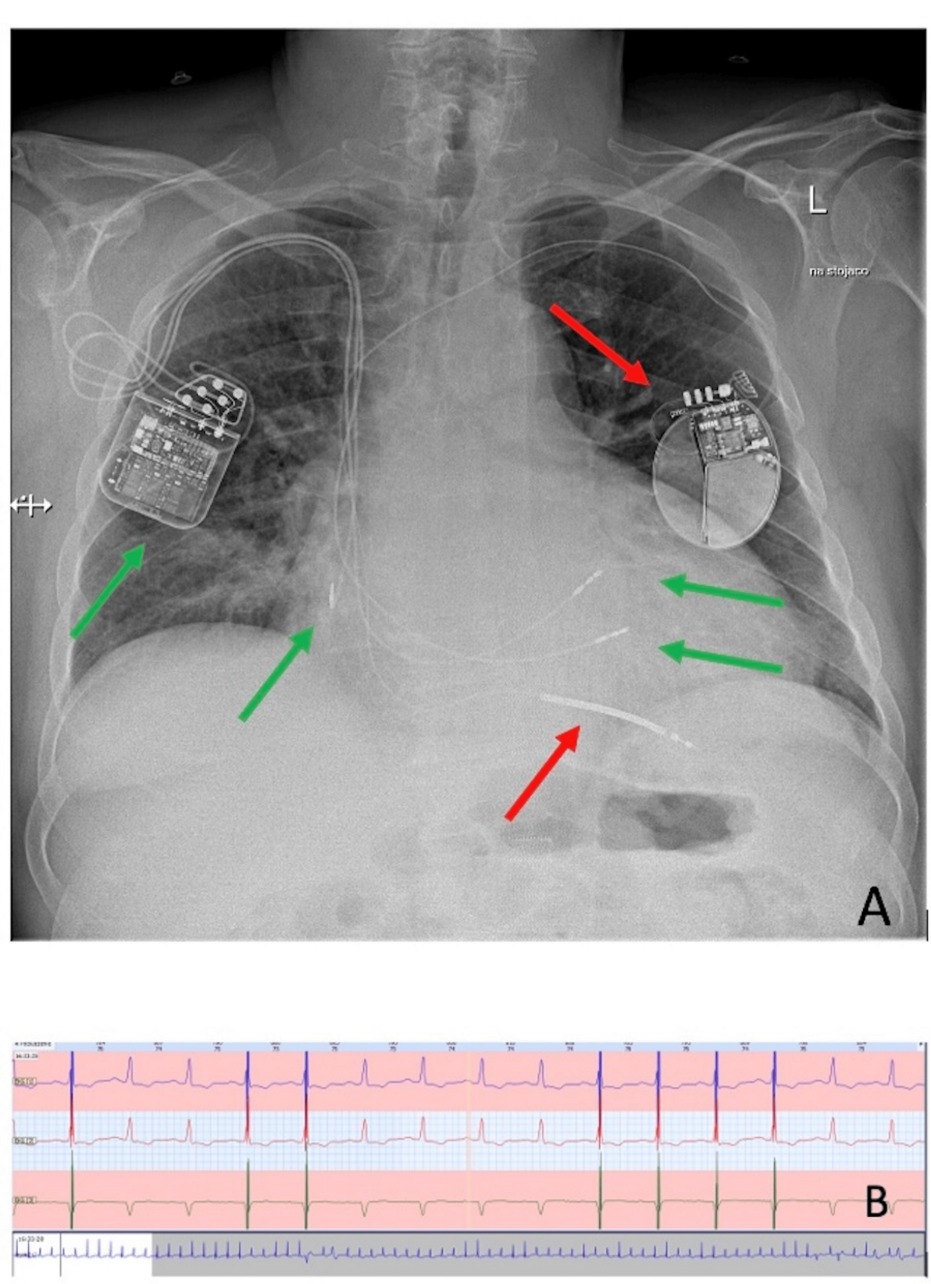
A. Chest radiograph: implantable cardioverter-defibrillator (ICD) with an ICD lead (red arrows) and the cardiac contractility modulation device with leads (green arrows). B. Holter monitoring: loss of cardiac contractility modulation.

In 2018, the patient underwent ICD implantation without evidence of device interference (Figure 3). In 2019, he reported a pulsatile sensation near the CCM pocket accompanied by clinical deterioration. Although device checks were initially unremarkable, reduction of impulse amplitude alleviated symptoms. Despite optimal pharmacotherapy, the patient’s condition worsened (NYHA III), with a decline in the six-minute walk distance (464 m to 150 m) and LVEF (to 26%).

**Figure 4:**
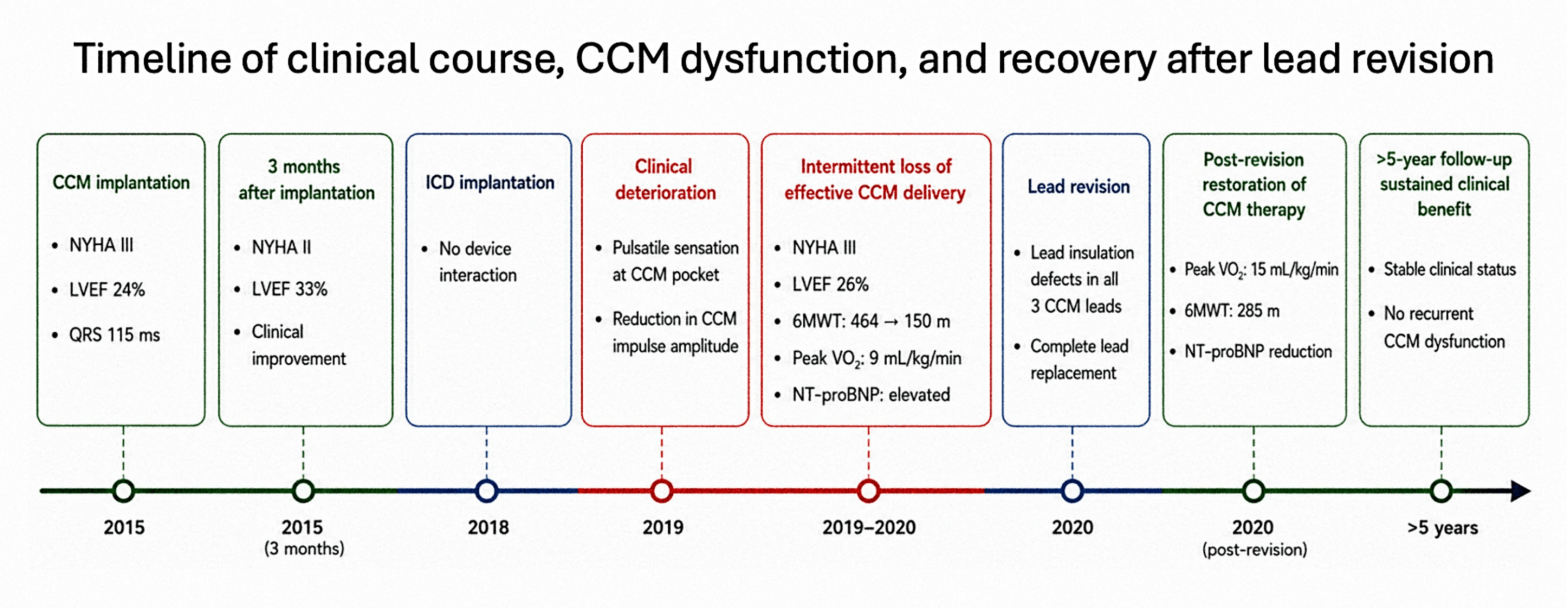
Timeline of the presented case CCM: Cardiac contractility modulation; NT-proBNP: N-terminal pro B-type natriuretic peptide; ICD: implantable cardioverter-defibrillator; NYHA: New York Heart Association The figure was created using Pixelmator Pro and Microsoft PowerPoint.

Serial ambulatory ECG recordings revealed intermittent loss of effective CCM delivery (Figure 4). Temporary deactivation of CCM resulted in further deterioration, and after exclusion of other causes, device dysfunction was suspected. In 2020, surgical revision revealed insulation defects in all three leads, which were replaced. Restoration of CCM function led to significant clinical improvement, including increased peak VO₂ (9 to 15 mL/kg/min), improved 6MWT (150 m to 285 m), and a reduction in N-terminal pro B-type natriuretic peptide levels. Clinical benefits have been sustained over five years.

## Discussion

This case highlights the critical role of effective CCM delivery in maintaining clinical stability. Device dysfunction, even in the absence of obvious abnormalities during routine interrogation, may lead to significant deterioration. Optimization of pharmacotherapy alone was insufficient, emphasizing the importance of addressing device-related issues. 

Previous reports have described marked responses to CCM, raising the possibility of “super-responders” [10]. Meta-analyses of randomized trials demonstrate that CCM improves quality of life and exercise capacity, although effects on mortality and hospitalization remain inconclusive. Greater benefit has been observed in selected subgroups, including patients with LVEF 25-45% [3,11].

Real-world data further support the clinical utility of CCM, showing reductions in HF-related hospitalizations and improvements in functional status [12,13]. Importantly, CCM does not appear to increase arrhythmogenic risk [3]. However, the presence of multiple intracardiac leads, particularly in patients with concomitant ICD systems, may increase the risk of lead-related complications [14].

In this case, CCM dysfunction was caused by lead insulation failure, likely due to mechanical interaction between leads. To our knowledge, this is the first reported case of CCM system revision due to lead failure leading to clinical deterioration. This finding underscores the need for careful long-term monitoring of device function beyond routine checks. Combining CCM therapy with a subcutaneous ICD or an extravascular ICD appears feasible and may help mitigate device-related complications associated with transvenous systems [15,16].

## Conclusions

This case illustrates that the clinical benefits of CCM may be highly dependent on continuous and effective therapy delivery. To our knowledge, this is the first report demonstrating HF deterioration caused by CCM lead failure with subsequent recovery following system revision. These findings expand the current evidence on CCM by highlighting the importance of considering device malfunction as a potential cause of otherwise unexplained clinical worsening and underscore the need for long-term monitoring of CCM system integrity. 
